# Neoadjuvant Chemoradiation Combined with Regional Hyperthermia in Locally Advanced or Recurrent Rectal Cancer

**DOI:** 10.3390/cancers13061279

**Published:** 2021-03-13

**Authors:** Oliver J. Ott, Cihan Gani, Lars H. Lindner, Manfred Schmidt, Ulf Lamprecht, Sultan Abdel-Rahman, Axel Hinke, Thomas Weissmann, Arndt Hartmann, Rolf D. Issels, Daniel Zips, Claus Belka, Robert Grützmann, Rainer Fietkau

**Affiliations:** 1 Department of Radiation Oncology, Universitätsklinikum Erlangen, 91054 Erlangen, Germany; manfred.schmidt@uk-erlangen.de (M.S.); thomas.weissmann@uk-erlangen.de (T.W.); rainer.fietkau@uk-erlangen.de (R.F.); 2 Department of Radiation Oncology, Universitätsklinikum Tübingen, 72076 Tübingen, Germany; cihan.gani@med.uni-tuebingen.de (C.G.); ulf.lamprecht@med.uni-tuebingen.de (U.L.); daniel.zips@med.uni-tuebingen.de (D.Z.); 3 Department of Medicine III, University Hospital, LMU Munich, 81377 Munich, Germany; lars.lindner@med.uni-muenchen.de (L.H.L.); abdel-rahman@med.uni-muenchen.de (S.A.-R.); rolf.issels@med.uni-muenchen.de (R.D.I.); 4 Cancer Clinical Research Consulting (CCRC), 40595 Düsseldorf, Germany; axel.hinke@hhu.de; 5 Institute of Pathology, Universitätsklinikum Erlangen, 91054 Erlangen, Germany; arndt.hartmann@uk-erlangen.de; 6 Department of Radiation Oncology, University Hospital, LMU Munich, 80377 Munich, Germany; claus.belka@med.uni-muenchen.de; 7 German Cancer Consortium (DKTK), 80336 Munich, Germany; 8 Department of Surgery, Universitätsklinikum Erlangen, 91054 Erlangen, Germany; robert.gruetzmann@uk-erlangen.de

**Keywords:** locally advanced rectal cancer, locally recurrent rectal cancer, concurrent chemoradiation, complete remission rate, tumor regression grading, regional hyperthermia

## Abstract

**Simple Summary:**

The HyRec trial was initially designed to optimize and standardize the treatment of locally recurrent rectal cancer (LRRC). An escalated neoadjuvant treatment schedule, consisting of curative radiotherapy, concurrent chemotherapy with 5-Fluorouracil and Oxaliplatin, and additional regional hyperthermia, was evaluated with the intention to increase the rate of curative resections. Primary endpoints were the feasibility rate defined by the number of therapy-limiting toxicity or treatment withdrawal, and the pathologically confirmed complete remission (pCR) rate. Between 2012 and 2018, 111 patients with Union for International Cancer Control (UICC) stage IIB-IV or any locally recurrent rectal cancer were included. The intensified neoadjuvant and multimodality treatment schedule was feasible and led to comparable early toxicity rates as described by other trials that used the similar chemoradiation protocol. The presented treatment regimen resulted in a very high pCR rate and appears as a promising option for patients with LRRC.

**Abstract:**

Background: To prospectively analyze feasibility and pathological complete response (pCR) rates of neoadjuvant chemoradiotherapy combined with regional hyperthermia (RHT) in patients with locally advanced (LARC) or recurrent (LRRC) rectal cancer. Methods: between 2012 and 2018, 111 patients with UICC stage IIB-IV or any locally recurrent rectal cancer were included (HyRec-Trial, ClinicalTrials.gov Identifier: NCT01716949). Patients received radiotherapy with concurrent 5-Fluororuracil (5-FU)/Capecitabine and Oxaliplatin, and RHT. Stage 1 feasibility analysis evaluated dose-limiting toxicities (DLT) after 19 patients, stage 2 after 59 evaluable patients. Analysis of the pCR rate was based on histopathological reports. Results: the feasibility rates for stages 1 and 2 were 90% (17/19) and 73% (43/59), respectively. In the intention-to-treat population the pCR rate was 19% (20/105; 90% confidence interval (CI) 13.0–26.5). In the per-protocol-analysis, complete tumor regression was seen in 28% (18/64) and 38% (3/8) of the patients with LARC and LRRC, respectively. Complete resection rates (R0) among patients with LARC and LRRC who received surgery were 99% (78/84) and 67% (8/12). Conclusions: the intensified neoadjuvant and multimodality treatment schedule was feasible and led to comparable early toxicity rates as described by other trials that used the similar chemoradiation protocol. The presented treatment regimen resulted in a very high pCR rate and appears as a promising option for patients with LRRC.

## 1. Introduction

The HyRec trial was initially designed to optimize and standardize the treatment of locally recurrent rectal cancer (LRRC). At the beginning of the 21th century, non-resectable and especially previously irradiated local rectal cancer recurrences were usually regarded as palliative cases. At this time, typical re-irradiation concepts administered another 30 Gy of radiation in order to slow down tumor growth. Other patients received various systemic treatments or intraoperative irradiation. On the other hand, microscopically complete resection (R0) was already known as a prerequisite for long-term cure, which could be reached in about 25–50% of the patients with LRRC [1]. Therefore, a protocol with a threefold escalated neoadjuvant treatment schedule was designed with the intention to increase the rate of curative resections. Firstly, the total radiation dose was increased to a curative level of 45–50.4 Gy, even for previously irradiated patients. Secondly, simultaneous chemotherapy was not 5-Fluorouracil (5-FU) alone, but complemented with Oxaliplatin. Alternative use of the oral prodrug Capecitabine instead of 5-FU was allowed. Thirdly, during the radiotherapy series, concurrent regional hyperthermia (RHT) was added based on existing data on higher response rates for the combination of radiotherapy and RHT compared to irradiation alone [2,3,4].

Concurrently, the discussion about omitting surgery in patients with a pathological complete response (pCR) after chemoradiation gained more and more attraction [5,6]. The question was whether the escalated neoadjuvant HyRec protocol could lead to higher pCR rates. Therefore, we decided to include primary rectal cancers as well and introduced the pCR rate as primary efficacy endpoint.

## 2. Patients and Methods

**Figure 1:**
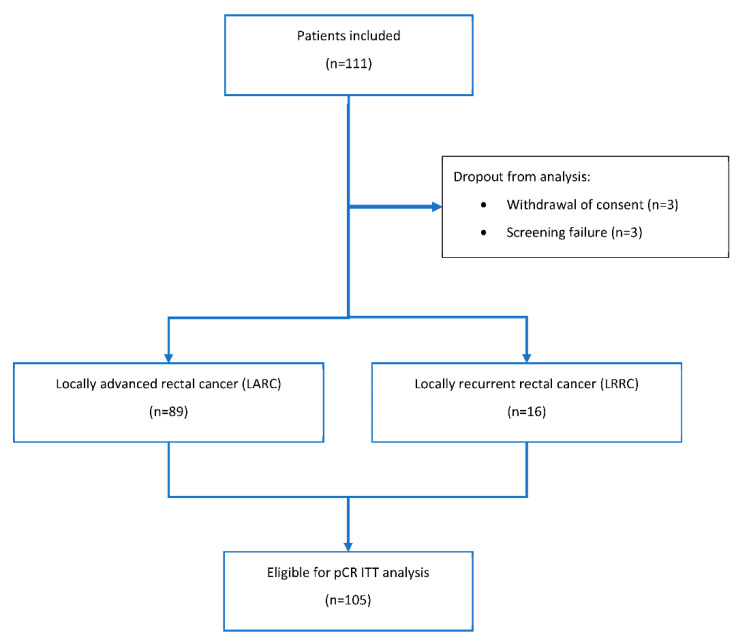
Consort diagram. pCR: pathologically confirmed complete remission. ITT: intention-to treat.

Between 2012 and 2018, 111 patients with histologically confirmed UICC stage IIB-IV adenocarcinoma of the rectum were enrolled. Six patients dropped out from primary intention-to-treat (ITT) efficacy analysis (see Figure 1). Finally, 89/105 (85%) of the patients had a locally advanced rectal cancer (LARC), and 16/105 (15%) a LRRC. All treatments described in the manuscript were carried out in accordance with national law and the Helsinki Declaration of 1975 in its current, revised form [7]. Informed consent was obtained from all patients (University of Erlangen Ethical Review Committee No. 119_12 Az).

Radiotherapy was applied using linear accelerators to deliver megavoltage external-beam irradiation to the primary and lymphatics or the local recurrence. For treatment planning, either 3D-conformal or intensity-modulated radiotherapy (IMRT) techniques were applied with 6 MV photon beams, at least. Dose was specified according to the International Commission on Radiation Units (ICRU) Reports 50 and 62 [8]. Regarding LARC, the planning target volume (PTV) encompassed the gross tumor volume (GTV) and the pelvic lymphatics (CTV) with an additional appropriate safety margin of 3–5 mm for intrafractional motion and daily interfractional positioning errors. Radiotherapy was administered once daily, five days a week, up to a total dose of 50.4 Gy (28 × 1.8 Gy). In cases of LRRC without prior pelvic irradiation, PTV definition and dose followed the same standards as for LARC. For patients with LRRC and prior pelvic radiotherapy in their histories, the target volume consisted of the GTV with a safety margin of 1–2 cm in each of six directions for covering both CTV and PTV with respect to non-infiltrated anatomical borders. These patients received daily radiotherapy fractions up to a total dose of 45 Gy (25 × 1.8 Gy). Further boost irradiation was not performed in any patient.

Patients received simultaneous chemotherapy with 5-FU and Oxaliplatin. 5-FU was applied with 250 mg/m^2^/d as continuous intravenous infusion on days 1–14 and 22–35. Oxaliplatin with 50 mg/m^2^ was given as intravenous bolus infusion diluted in 500 mL glucose 5% over two hours on days 2, 9, 23, and 30. Alternatively, 5-FU could have been replaced by its prodrug Capecitabine with oral doses of 1650 mg/m^2^/d on days 1–14 and 22–35, given in two separate doses of 825 mg/m^2^, in the morning and evening.

RHT was applied in accordance with the published guidelines for the use within clinical studies [9,10]. It was performed with the BSD 2000-3D- and BSD 2000-3D-MR-Hyperthermia Systems^TM^ (BSD Medical Corporation/Pyrexar, Salt Lake City, UT, USA) using either the SigmaEye^TM^, SigmaEye-MR^TM^, or Sigma60^TM^ applicator, depending on the abdominal diameter of the patient. RHT was given twice weekly and started right before irradiation up to ten treatments. The interval between two RHT treatments was 72 h at least. Thermometry probes were inserted in the rectum, the bladder, the vagina, and the rima ani for continuous thermometry and thermal mapping. Therapeutic time started when the tumor-related temperature in the rectum reached a minimum of 41.5 °C or 30 min after enabling power. Therapeutic time was scheduled to be 60 min, the maximum total duration was limited 90 min. During treatment, patients’ cardiac function was continuously monitored by electrocardiogram, and blood pressure and oxygen saturation levels were constantly controlled.

Surgery was performed 4–8 weeks after chemoradiation. All surgical procedures had been performed according to the institutional standards in consideration of contemporary guidelines. Per protocol, surgery was not regarded as an integral part of the tested study regimen.

The baseline assessment included history taking, physical examination, quality of life questionnaire, electrocardiogram, pregnancy test, extensive hematological tests, baseline toxicity grading, and staging examinations (histology, chest X-ray, endosonography, rectosigmoideoscopy/coloscopy, abdominal computed tomography). The weekly assessments during therapy included physical examination, hematological tests, and toxicity assessment according to the National Cancer Institute Common Terminology Criteria for Adverse Events (NCI CTCAE) v.4.0. Six weeks after chemoradiotherapy (CRT), the weekly assessments were repeated (’the end of treatment visit’).

Follow-up examinations were scheduled 3, 6, 12, 18, 24, 36, 48, and 60 months after the end of treatment visit and included histological results, physical examination, and a toxicity assessment according to NCI CTCAE v.4.0 with respect to late complications, and tumor status assessments. Pathological assessment was designed in analogy to our institutional precursor trial [11] and performed in accordance to the applicable national guideline. Minimal requirements for pathological evaluation were histopathologic tumor type, pT-category, pN-category, number of nodes, grading, marginal distances, and R-category. We have previously shown that tumor regression grading (TRG), a semiquantitative assessment of residual tumor cells versus fibroinflammatory tissue in the rectal wall, was able to stratify tumor response to chemoradiation and predict prognosis on an individual-patient [12]. In this study, TRG was recorded prospectively according to Dworak et al. [13]. If surgery was performed, the Dworak TRG score was performed by the same pathologist as in the study of Fokas et al. [12] in the majority of the samples. Departmental four-eye-confirmation of pathological results was a standard procedure during the trial.

Considering a proportion of therapy-limiting toxicity or treatment withdrawal of up to 15% as feasible, but a proportion of 30% or more as a clear sign of insufficient feasibility, 59 patients were required to achieve 80% power on a type-one error level of 0.05. The applied two-step design according to Simon [14] allowed for early stopping for futility after an interim analysis of the first 19 patients. Once feasibility was established, the trial was amended in order to obtain evidence for a superior efficacy (pCR rate of 20% or more) in contrast to an assumed pCR of merely 10% after standard therapy, based on historical data [2,4,15,16]. This required 102 evaluable patients (110 allowing for dropouts) in a single-step phase II design achieving 90% power with a type-one error level of 0.05.

Survival type endpoints were analyzed using the Kaplan–Meier method. The reported p-values are generally two-sided and considered to be explorative.

## 3. Results

The median age of all patients was 58 (range; 23–85) years. Males were much more frequent (82/105; 78%), especially in the LRRC group (15/16; 94%). The Eastern Co-operative of Oncology Group (ECOG) performance score was 0–1 in 104/105 patients (99%). A detailed description of patient, disease, and treatment characteristics may be found in Table 1.

### 3.1. Protocol Treatment Adherence

Radiotherapy was well-tolerated. The mean treatment duration was 38 days (95% confidence interval (CI): 37.0–38.1) for all patients; 38 days (95% CI: 37.5–38.6) and 35 days (95% CI: 33.4–36.2) for patients with LARC and LRRC, respectively. Nearly all patients (104/105) received the scheduled irradiation dose; one LARC patient got a dose reduction to 45 Gy because of diarrhea. Eight percent of the patients (8/105) experienced a radiotherapy delay, mainly because of administrative reasons. The median delay was 2 days (range; 2–6) and 1 day for patients with LARC and LRRC, respectively.

The mean number of RHT fractions was 9.0 (95% CI: 9.0–9.6) treatments in LARC and 9.3 (95% CI: 8.4–10.2) in LRRC. Ninety percent of the patients (94/105) received seven RHT fractions, at least.

Concurrent chemotherapy with 5-FU/Capecitabine and Oxaliplatin was well-tolerated in the vast majority of patients. More detailed information on treatment variables and protocol violations may be found in Table 1.

### 3.2. Stage 1 Feasibility Analysis

Of the first 20 patients that entered this analysis, one patient dropped out because of a screening failure. Among the remaining 19 patients, 14/19 (74%) had a LARC and 5/19 (26%) a LRRC. No grade 4–5 adverse events occurred. No leukopenia or neutropenia of grade 3 with complications such as fever (>38.5 °C) or infection, or with a duration of >7 days was found. Two patients experienced a non-hematological toxicity of grade 3. In any patient, no toxicity led to permanent discontinuation of at least one of the drugs or other treatment modalities or a delay of treatment of more than three weeks. All patients received ≥ 70% of the scheduled hyperthermia applications. In summary, 2/19 (11%) fulfilled the dose-limiting-toxicity (DLT) criteria, which corresponded with a feasibility rate of 90%.

### 3.3. Stage 2 Feasibility Analysis

Per protocol, 59 patients had to be recruited for the second stage. Because of six dropouts (see Figure 1) 59 of 65 patients enrolled qualified for this analysis; 47/59 (80%) with a LARC and 12/59 (20%) with a LRRC. No grade 4–5 adverse events occurred. No leukopenia or neutropenia of severity grade 3 with complications as mentioned above was found. Fourteen of 59 patients (24%) experienced a non-hematological toxicity of grade 3. In 5/59 patients (9%), toxicity led to permanent discontinuation of at least one of the drugs or other treatment modalities or a delay of treatment of more than three weeks. A total of 55/59 (93%) patients received ≥ 70% of the scheduled hyperthermia applications. A comprehensive case-based presentation of the dose limiting toxicities may be found in Table 2. In summary, 16/59 (27%) experienced a DLT, which corresponds to a feasibility rate of 73%.

### 3.4. Primary Efficacy Endpoint pCR Rate und Tumor Regression Grading

In an amendment from 2015, the pathologic complete response (pCR) rate was chosen as primary efficacy endpoint. Between 2012 and 2018, 111 patients were included. Because of six dropouts (for details, see Figure 1), 105 patients entered the intention-to-treat (ITT) analysis. Among the 105 patients 71/105 (68%) had no pCR, 11/105 (11%) had no curative surgery, and in three cases (3%) data on the remission status was not available. Six out of eleven cases without curative surgery had a LRRC, of whom three had a poor response to the neoadjuvant treatment and three others with initially present distant metastasis had progressive metastatic disease. Three of five patients with LARC without surgery also had progressive distant disease with metastasis present at the time of study inclusion. The two others refused curative surgery because of a clinical complete response. One of them was tumor-free at the last follow-up visit 16 months after study inclusion; the other experienced a local recurrence after a follow-up of 56 months. Regarding the ITT analysis, the proportion with pCR was 20/105 (19%; 90% CI 13.0–26.5) among all patients. Thus, as the lower limit of the one-sided interval excludes the futility threshold of 10% with 95% confidence the study is formally positive with respect to the efficacy endpoint. The pCR rates for patients with LARC and LRRC were 17/89 (19%) and 3/16 (19%), respectively. Excluding the patients with initially diagnosed distant metastases, the proportion with pCR was 19/95 (20%) among all patients, and 16/84 (19%) and 3/11 (27%) for patients with LARC and LRRC, respectively.

Additionally, the tumor regression grading (TRG) according to Dworak [13] was determined, in case curative surgery was performed. The Dworak TRG score considers the response of the primary tumor exclusively. Therefore, one additional patient with LARC (postsurgical TNM staging: ypT0ypN1b) was rated as Dworak TRG 4. The score was not provided by all participating centers’ pathologists and available in 72 patients. In the per-protocol analysis of the available cases, a Dworak TRG 4 score was found in 28% (18/64) and 38% (3/8) of the patients with LARC and LRRC, respectively. A combined Dworak 3–4 score as expression of an at-least subtotal complete remission was found in 49/72 (68%) patients. A detailed summary of the Dworak TRG analysis may be found in Table 3.

### 3.5. Secondary Endpoints

**Figure 2:**
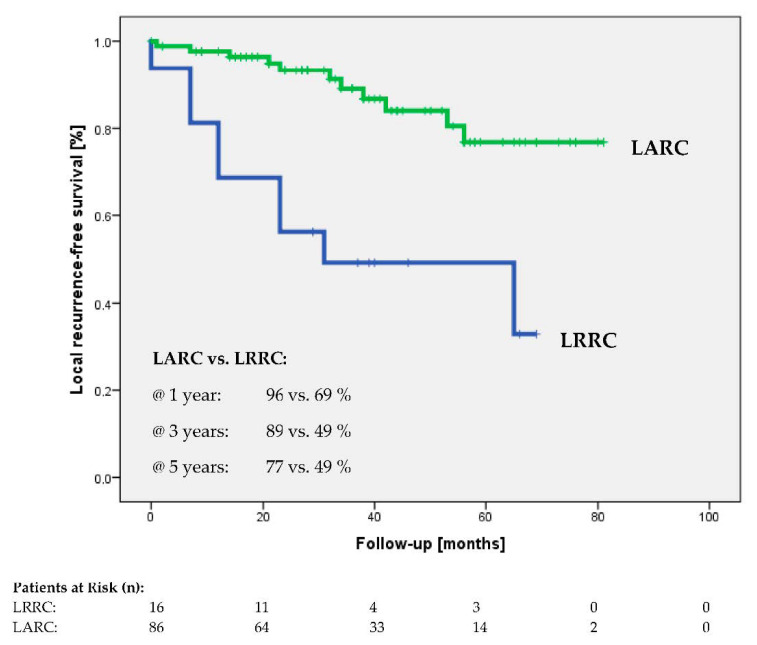
Local recurrence-free survival of locally advanced (LARC) and locally recurrent (LRRC) rectal cancer.

Median follow-up for all patients was 34 months (range; 0–81). Five-year overall survival was 75% for the whole group, and 82 vs. 46% for patients with LARC and LRRC. At the time of analysis, 16/105 (15%) patients were dead, 10/105 (9.5%) experienced local recurrence, and 35/105 (33%) had distant metastases. Five-year local recurrence-free and distant metastasis-free survival rates were 77 vs. 49% (see Figure 2) and 60 vs. 41%, respectively. Nine of sixteen patients (56%) died without local control and 15/16 (94%) with distant metastases, and all 16 patients died because of the tumor disease. Regarding LRRC patients only, 7/7 died without local control and 6/7 with distant metastases, and exclusively regarding LARC patients 2/9 died without local control and 9/9 with distant metastases. Disease-specific (DSS) and disease-free survival (DFS) rates differed between the subgroups at five years: 85 vs. 52% and 57 vs. 37%. Among the curatively operated patients, the rates of microscopically complete resections (R0) were 78/79 (99%) and 10/12 (80%) for the LARC and LRRC groups, respectively.

### 3.6. Adverse Events

No grade 4–5 early toxicities occurred. A hematotoxic event grade 3 was detected in 11/105 (11%) patients; non-hematotoxic side effects grade 3 were found in 29/105 (28%) cases. During the course of the trial, 20 serious adverse events (SAEs) were recorded and recovered in 17/20 cases at the time of analysis. A comprehensive overview of early toxicity is given in Table 4. While focusing on the primary endpoints, feasibility and tumor regression, late toxicity was not part of the present analysis.

## 4. Discussion

The present study is the largest prospective study of regional hyperthermia in the context of chemoradiation for rectal cancer. The target population for inclusion were patients with an unfavorable prognosis, reflected by the inclusion of patients with LRRC and exclusion of primary T3N0 disease. Moreover, in the subgroup with LRRC, 31% of the patients had oligometastases and more than half of them had been previously treated with radiotherapy because of rectal cancer. Pelvic re-irradiation is a very challenging scenario since applicable radiation doses are limited due to the preceding normal tissue dose burden. Prognosis is limited and a poor three-year overall survival rate of 38% was reported in a recent meta-analysis [17]. In this setting, additional RHT is a very promising tool to compensate for the limited radiation dose applicable [2,3,4,15,18]. This has already been demonstrated for other tumor entities such as breast cancer [19]. For LRRC, it has repeatedly been shown that patients who undergo surgery after neoadjuvant radiotherapy show superior survival compared to patients who turn out to be ineligible for surgery [20]. In our trial, 63% of the patients with LRRC underwent curative surgery, the majority of those with negative margins, which can be interpreted as a function of the intensified local treatment. The three-year overall survival of the patients with LRRC was 85%. The same holds true for local control. Compared with data from studies on LRRC treated without hyperthermia, these numbers appear very promising. For instance, in a report from the Netherlands, a three-year overall survival of approximately 50% is reported and 46% of the patients achieved R0 resection after re-irradiation and surgery [21]. Selvaggi et al. reported similar values in multicenter analysis of 150 patients with LRRC [22]. In their metaanalysis on LRRC, Tanis et al. reported on 55 cohort studies comprising 3767 patients with a special interest on the rate of intentionally curative treatments performed [1]. The microscopically complete resection rate (R0) was 56%, and overall survival ranged between 25 to 41%. This means that long-term cure may be reachable even for patients with LRRC when a curative resection could be performed. Regarding patients with LARC, it should be noted that patients with early UICC stage II tumors (T3N0) were not eligible for inclusion, since many of these patients already have a very favorable prognosis with a low risk of distant metastases. Instead, with 21% of the cases the rate of patients with cT4 tumors was more than twice as high as in a recent meta-analysis of major landmark trials of neoadjuvant radiochemotherapy in rectal cancer [23], which should be taken into account for the interpretation of our results.

The chemoradiation protocol used was firstly described by Rödel et al. [11]. In their multicenter feasibility phase-2 trial, radiotherapy was applied as prescribed in 95/104 (91%) patients [11]. Unplanned irradiation treatment interruptions of more than two days because of toxicity occurred in five patients (5%). In four patients (4%), radiotherapy was discontinued because of diarrhea, ileus, and genitourinary toxicity. In the randomized German Rectal Cancer Study Group CAO/ARO/AIO-04 Trial, the similar chemoradiation protocol was applied for the experimental arm [24]. Full-dose radiotherapy was administered in 571/606 (94%), with interruptions in 59/606 (10%), and discontinued prematurely in 19/606 (3%) of the patients. In comparison, the results of the HyRec trial were quite similar. Only one patient with a LARC was discontinued at 45 Gy because of diarrhea. In the CAO/ARO/AIO-04 Trial, concurrent full dose chemotherapy with 5-FU and Oxaliplatin was administered in 85% (516/606) [24]. In the HyRec trial, full-dose concurrent radiotherapy was applied in 81/105 (77%); 15/105 (14%) had a dose reduction because of toxicity, and 7/105 (7%) had only a very slight dose reduction because of administrative reasons. The compliance with hyperthermia cannot compared to other trials, because the study schedule with 2 hyperthermia fractions per week up to a total of 10 was introduced for the first time ever. The median number of RHT treatments given was very high, 94/105 (90%) of the patients received seven fractions, at least (see Table 1). In summary, compliance with the protocol treatment was regarded as good with no significantly deviating results in comparison to previously published data [24,25]. In comparison to the results of the German Rectal Cancer Study Group CAO/ARO/AIO-04 Trial [24], early toxicity was quite similar with a slightly lower rate of gastrointestinal toxicity (15 vs. 20%) and a slightly higher rate of grades 3–4 radiodermatitis (7 vs. 2%) and grades 2–3 neuropathy (7 vs. 2%) for the HyRec Trial. The stage 1 and 2 feasibility analyses underlined that the presented escalated multimodal study design was formally feasible.

With a global pCR rate of 19% and a Dworak TRG score 3–4 in 68% of the evaluable patients’ tumor regression was very high, particularly regarding the unfavorable patient selection in our trial. These results are of highest clinical relevance. In the context of organ preservation strategies, the development of radiotherapy protocols that increase clinical complete response rates with limited toxicity and thus quality more patients for a “watch and wait” approach, are in the focus of research [26]. According to the standards of the time when the trial was initiated, surgery was scheduled four to six weeks after the end of radiotherapy. There is growing evidence showing that the highest pathological complete response rates are achieved with an interval of approximately ten weeks [23]. It therefore appears likely that some patients with a strong but not complete tumor regression would have achieved a complete response with a longer time between neoadjuvant treatment and surgery. Regarding pathological response and long-term oncological outcomes, previous retrospective trials had shown promising results [15,18]. With a local and distant control of 96% and 77%, respectively, after three years, we were able to prospectively confirm this data. The results are comparable or even superior to data from non-hyperthermia trials that also included early stage-II tumors [27,28]. However, some limitations of our study have to be addressed. The subgroup of LRRC was prospective but small as it is in the majority of published reports. Furthermore, patients with severe cardiac comorbidities were ineligible for hyperthermia, which might introduce a bias in terms of patient fitness.

Currently the field of perioperative treatment of rectal cancer is subject to major dynamics. Total neoadjuvant therapy with the administration consolidation or induction chemotherapy is discussed as a potential new standard of care. The ongoing CAO/ARO/AIO-16 trial is currently investigating the potential of this approach in order to maximize complete response rates (ClinicalTrials.gov Identifier: NCT03561142). In this trial, hyperthermia is applied as a local intensification of treatment as well. Recently the RAPIDO trial has shown a significantly improved disease related treatment failure rate with total neoadjuvant therapy consisting of short-course radiotherapy and followed by 18 weeks of systemic treatment [29]. However, the local failure rate in the experimental arm was still 8.7%, pointing to the need for further optimization of local treatment.

## 5. Conclusions

The intensified neoadjuvant and multimodality treatment schedule with full-dose external beam radiotherapy, concurrent 5-FU/Capecitabine and Oxaliplatin, and additional RHT was feasible and led to comparable early toxicity rates as described by other trials that used the similar chemoradiation protocol. The presented treatment regimen resulted in a very high pCR rate and tumor regression and appears as a promising option for patients with LRRC.

## Figures and Tables

**Table 1:** Patient-, disease-, and treatment-related characteristics.

Characteristics	Locally Advanced Rectal Cancer	Locally Recurrent Rectal Cancer (LRRC, *n* = 16)
	(LARC, *n* = 89)	
Initial clinical T-category (n/N [%])		
T2/rT2	6/89 (7)	1/16 (6)
T3/rT3	63/89 (71)	7/16 (44)
T4/rT4	19/89 (21)	8/16 (50)
n.a.	1/89 (1)	-
Initial clinical N-category (n/N [%])		
N0/rN0	6/89 (7)	11/16 (69)
N1/rN0	46/89 (52)	2/16 (12)
N2/rN2	36/89 (40)	3/16 (19)
n.a.	1/89 (1)	-
Initial M-category (n/N [%])		
M0	84/89 (94)	11/16 (69)
M1	4/89 (5)	5/16 (31)
n.a.	1/89 (1)	-
Previous oncological treatments (n/N [%])		
surgery	1/89 (1)	15/16 (94)
radiotherapy	-	10/16 (63)
chemotherapy	-	12/16 (75)
External beam radiotherapy		
median total dose [Gy]	50.4 (45.0–50.4)	45.0 (45.0–50.4)
median single dose [Gy]	1.80 (-)	1.80 (-)
radiotherapy delay (n/N [%]) *	5/88 (6)	3/16 (19)
median duration [days]	2 (2–6)	1 (-)
total dose reduction (n/N [%]) *	1/88 (1)	-
premature termination *	1/89 (1)	-
Chemotherapy (n/N [%])		
schedule		
5-FU and oxaliplatin	81/89 (91)	12/16 (75)
capecitabine and oxaliplatin	7/89 (8)	3/16 (19)
5-FU/capecitabine and oxaliplatin	1/89 (1)	1/16 (6)
5-FU protocol adherence		
administered per protocol	56/81 (69)	11/12 (92)
interval prolongation *	14/81 (17)	1/12 (8)
dose reduction *	11/81 (14)	1/12 (8)
premature termination *	2/81 (2)	-
n.a.	2/81 (2)	-
capecitabine protocol adherence		
administered per protocol	3/7 (43)	2/3 (67)
interval prolongation *	3/7 (43)	1/3 (33)
dose reduction *	3/7 (43)	1/3 (33)
premature termination *	3/7 (43)	-
oxaliplatin protocol adherence		
administered per protocol	58/89 (87)	13/16 (81)
interval prolongation*	17/89 (19)	2/16 (13)
dose reduction *	9/89 (10)	1/16 (6)
premature termination *	6/89 (7)	1/16 (6)
n.a.	2/89 (2)	-
Regional hyperthermia (RHT)		
median number of fractions [n]	10 (1–11)	10 (4–10)
≥7 fractions (n/N [%])	80/88 (91)	14/16 (88)
mean total treatment time [min]	837 ± 142	831 ± 170
mean CEM43 °C [min]	6.4 ± 5.2	±4.9

LARC: locally advanced rectal cancer, LRRC: locally recurrent rectal cancer. 5-FU: 5-fluorouracil; *: multiple responses possible; n.a.: data not available; CEM43 °C: cumulative equivalent minutes at 43 degree Celsius. RHT: regional hyperthermia.

**Table 2:** Case-related dose-limiting toxicities (DLT).

Cases	Allergic Reaction	Proctitis	Pain	Radiodermatitis	Nausea	Others	Discontinuation Radiotherapy	Discontinuation 5-FU	Discontinuation Oxaliplatin	Discontinuation Hyperthermia
01010		x	x	x		x				x *
02001			x							
01014			x			x				
01019						x				x
01020			x							
01023				x						
01026							x		x	
01028		x		x						
01030		x								
01031						x				
01035						x			x	x
01034				x						
01036										x *
01037	x									
01039					x	x		x	x	
01041				x						

* Patient’s request. 5-FU: 5-Fluorouracil.

**Table 3:** Tumor regression grading after curative resection according to Dworak [13].

TRG score	All Patients (*n* = 94)	LARC (*n* = 84)	LRRC (*n* = 10)
Dworak 1	7/94 (7)	5/84 (6)	2/10 (20)
Dworak 2	16/94 (17)	14/84 (17)	2/10 (20)
Dworak 3	28/94 (30)	27/84 (32)	1/10 (10)
Dworak 4	21/94 (22)	18/84 (21)	3/10 (30)
n.a. *	22/94 (23)	20/84 (24)	2/10 (20)

*: the Dworak TRG score was not provided by all participating centers’ pathologists. LARC: locally advanced rectal cancer, LRRC: locally recurrent rectal cancer.

**Table 4:** Adverse events according to NCI CTCAE v.4.0.

Adverse Event	Grade 1–2 n/N (%)	Grade 3 n/N (%)	N.a. n/N (%)
Anemia	71/105 (68)	1/105 (1)	6/105 (6)
Leucopenia	44/105 (42)	1/105 (1)	6/105 (6)
Neutropenia	6/105 (6)	-	6/105 (6)
Neutropenic fever	-	1/105 (1)	6/105 (6)
Thrombocytopenia	38/105 (36)	-	6/105 (6)
Elevated creatinine	15/105 (14)	-	6/105 (6)
Elevated bilirubine	12/105 (11)	-	6/105 (6)
Elevated transaminases (AST/ALT)	41/105 (39)	2/105 (2)	6/105 (6)
Elevated alkaline phosphatase	19/105 (18)	-	6/105 (6)
Aconuresis	4/105 (4)	-	7/105 (7)
Allergic reaction	11/105 (10)	2/105 (2)	7/105 (7)
Alopecia	5/105 (5)	-	7/105 (7)
Anal incontinence	29/105 (28)	1/105 (1)	7/105 (7)
Diarrhea	74/105 (70)	10/105 (10)	7/105 (7)
Dyspnea	9/105 (9)	-	7/105 (7)
Emesis	15/105 (14)	-	7/105 (7)
Erectile dysfunction	9/82 (11)	1/82 (1)	5/82 (6)
Fatigue	67/105 (64)	-	7/105 (7)
Fever	15/105 (14)	-	7/105 (7)
Hand-foot-syndrome	7/105 (7)	-	7/105 (7)
Heart disorder	5/105 (5)	-	7/105 (7)
Hemorrhage	50/105 (48)	1/105 (1)	7/105 (7)
Mucositis	18/105 (17)	1/105 (1)	7/105 (7)
Nausea	39/105 (37)	2/105 (2)	7/105 (7)
Non-infectious cystitis	53/105 (50)	2/105 (2)	7/105 (7)
Obstipation	39/105 (37)	-	7/105 (7)
Pain	56/105 (53)	4/105 (4)	7/105 (7)
Peripheral motoric neuropathy	8/105 (8)	-	7/105 (7)
Peripheral sensoric neuropathy	64/105 (61)	-	7/105 (7)
Proctitis	62/105 (59)	3/105 (3)	7/105 (7)
Radiodermatitis	71/105 (68)	7/105 (7)	7/105 (7)
Urge to urinate	40/105 (38)	-	7/105 (7)
Vaginal stenosis	3/23 (13)	-	-
Weight loss	23/105 (22)	-	7/105 (7)
Other non-hematological AEs	20/105 (19)	7/105 (7)	6/105 (6)

N.a.: not available. AEs: adverse events. AST: glutamyl oxaloacetic transaminase/aspartate aminotransferase; ALT: glutamyl pyruvic transaminase/alanine aminotransferase.

## Data Availability

The data presented in this study are available on request from the corresponding author. The data are not publicly available due to privacy and ethical reasons.
